# Directed Data-Processing Inequalities for Systems with Feedback

**DOI:** 10.3390/e23050533

**Published:** 2021-04-26

**Authors:** Milan S. Derpich, Jan Østergaard

**Affiliations:** 1 Department of Electronic Engineering, Universidad Técnica Federico Santa María, Av. España 1680, Valparaíso 2390123, Chile; 2 Department of Electronic Systems, Aalborg University, 9220 Aalborg, Denmark

**Keywords:** data-processing inequality, directed information, mutual information, networked control, feedback capacity

## Abstract

We present novel data-processing inequalities relating the mutual information and the directed information in systems with feedback. The internal deterministic blocks within such systems are restricted only to be causal mappings, but are allowed to be non-linear and time varying, and randomized by their own external random input, can yield any stochastic mapping. These randomized blocks can for example represent source encoders, decoders, or even communication channels. Moreover, the involved signals can be arbitrarily distributed. Our first main result relates mutual and directed information and can be interpreted as a law of conservation of information flow. Our second main result is a pair of data-processing inequalities (one the conditional version of the other) between nested pairs of random sequences entirely within the closed loop. Our third main result introduces and characterizes the notion of in-the-loop (ITL) transmission rate for channel coding scenarios in which the messages are internal to the loop. Interestingly, in this case the conventional notions of transmission rate associated with the entropy of the messages and of channel capacity based on maximizing the mutual information between the messages and the output turn out to be inadequate. Instead, as we show, the ITL transmission rate is the unique notion of rate for which a channel code attains zero error probability if and only if such an ITL rate does not exceed the corresponding directed information rate from messages to decoded messages. We apply our data-processing inequalities to show that the supremum of achievable (in the usual channel coding sense) ITL transmission rates is upper bounded by the supremum of the directed information rate across the communication channel. Moreover, we present an example in which this upper bound is attained. Finally, we further illustrate the applicability of our results by discussing how they make possible the generalization of two fundamental inequalities known in networked control literature.

## 1. Introduction

The data-processing inequality states that if x,y,z are random variables such that x and z become independent when conditioning upon y, then:

(1) $$\begin{array}{c} I(x;y)\geq I(x;z) \end{array}$$

(2) $$\begin{array}{c} I(y;z)\geq I(x;z), \end{array}$$

where I(x;y) denotes the mutual information between x and y [1] (p. 252) (a definition of mutual information is provided in Section 2.2 below). Among its many uses, the data-processing inequality plays a key role in the proof of the converse part (i.e., outer bounds) in rate-distortion [1,2,3,4,5] (p. 317), channel capacity [1,6,7,8] (pp. 208, 217, 540 and 566), and joint source-channel coding theorems [1,9,10,11] (p. 221), and has recently been extended to von Newmann algebras, which have applications in quantum field theory (see [12] and the references therein).

**Figure 1:**
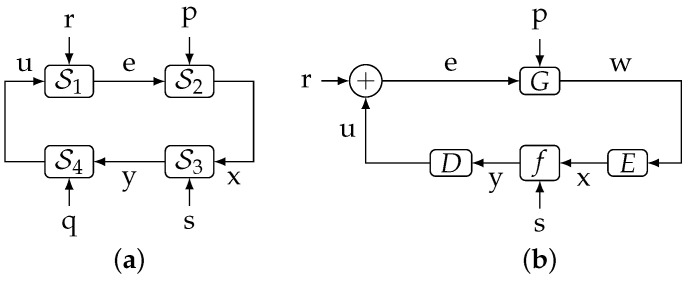
(**a**): The general system considered in this work. (**b**): A special case of (**a**), corresponding to the closed-loop system studied in [18].

It is well known that mutual information has an important limitation in systems with feedback, such as the one shown in Figure 1a. In this system, p, q, r, s, e, u, x, and y are random sequences, and the blocks $S_{1},\ldots ,S_{4}$ are deterministic causal mappings with an added delay of at least one sample. These blocks, randomized by their exogenous random inputs p, q, r, s, may yield any causal stochastic dynamic mappings. As pointed out in [13], for sequences inside the loop, such as x and y, I(x;y) does not distinguish the probabilistic interdependence produced by the effect x has on y from that stemming from the influence of y on x. This limitation motivated the introduction of the directed information in [13]. This notion assesses the amount of information that causally “flows” from a given random and ordered sequence to another. For this reason, it has increasingly found use in diverse applications, including characterizing the capacity of channels with feedback [13,14,15,16], the rate distortion function under causality constraints [5], establishing some of the fundamental limitations in networked control [17,18,19,20,21,22,23], determining causal relationships in neural networks [24], and portfolio theory and hypothesis testing [25], to name a few.

The directed information from a randomsequence $x^{k}$ to a random sequence $y^{k}$ is defined as:

(3) $$\begin{array}{c} I\left(x^{k}\to y^{k}\right)≜\sum_{i=1}^{k}I\left(y\left(i\right);x^{i}|y^{i-1}\right), \end{array}$$

where the notation $x^{i}$ represents the sequence x(1),x(2),…,x(i) and I(x;y|z) is the mutual information between x and y conditioned on (or given) z (hereafter we use non-italic letters, such as x, for random variables, denoting a particular realization by the corresponding italic character, *x*). The causality inherent in this definition becomes evident when comparing it with the mutual information between $x^{k}$ and $y^{k}$, given by $I\left(x^{k};y^{k}\right)=\sum_{i=1}^{k}I\left(y\left(i\right);x^{k}|y^{i-1}\right)$. In the latter sum, what matters is the amount of information about the *entire* sequence $x^{k}$ present in y(i), given the past values $y^{i-1}$. By contrast, in the conditional mutual information in the sum of (3), only the past and current values of $x^{k}$ are considered, that is, $x^{i}$. Thus, $I(x^{k}\to y^{k})$ represents the amount of information causally conveyed from $x^{k}$ to $y^{k}$. A related notion is the causally conditioned directed information introduced in [14], defined as:

(4) $$\begin{array}{c} I\left(x^{k}\to y^{k}\|q^{k}\right)≜\sum_{i=1}^{k}I\left(y\left(i\right);x^{i}|y^{i-1},q^{i}\right). \end{array}$$

In this paper, we derive inequalities involving directed and mutual information within feedback systems. For this purpose, we consider the general feedback system shown in Figure 1a. In this diagram, the blocks $S_{1},\ldots ,S_{4}$ represent possibly non-linear and time-varying causal discrete-time systems such that the total delay of the loop is at least one sample. These blocks can model, for example, source encoders, decoders or even communication channels. In the same figure, r,p,s,q are exogenous random signals (scalars, vectors, or sequences), which could represent, for example, any combination of disturbances, noises, random initial states, or side information. We note that any of these exogenous signals, in combination with their corresponding deterministic mapping $S_{i}$, can also yield any desired stochastic causal mapping (for example, a noisy communication channel, a zero-delay source coder or decoder, or a causal dynamic system with disturbances and a random initial state).

### 1.1. Main Contributions

**Figure 2:**
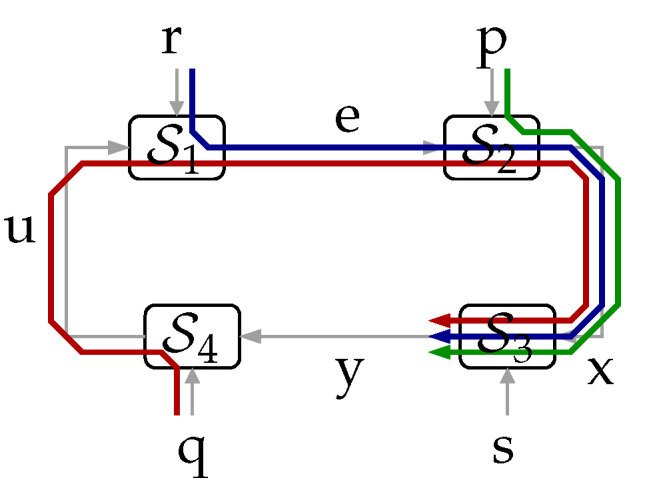
The flow of information between exogenous signals (p,q,r) and the internal signal y equals the directed information from $x^{k}$ to $y^{k}$ when s⫫(p,q,r).

1. Our first main contribution is the following theorem. It states a fundamental result, which relates the directed information between two signals within a feedback loop, say x and y, to the mutual information between an external set of signals and y:
  **Theorem** **1.** *In the system shown in Figure 1a, it holds that:*
  (5) $$\begin{array}{c} I\left(x^{k}\to y^{k}\right)=I\left(q^{k},r^{k},p^{k}\to y^{k}\right)-I\left(q^{k},r^{k},p^{k}\to y^{k}\|x^{k}\right)\leq I\left(p^{k},q^{k},r^{k}\;;y^{k}\right),\;\;\;\;\forall k\in N, \end{array}$$
  *with equality achieved if *s* is independent of (p,q,r).*
  The proof is in Section 3. This fundamental result, which for the cases in which s⫫(p,q,r) can be understood as a *law of conservation of information flow*, is illustrated in Figure 2. (Here, and in the sequel, we use the notation x⫫y to mean “x is independent of y”.) For such a cases, the information causally conveyed from x to y equals the information flow from (q,r,p) to y. When (p,q,r) are not independent of s, part of the mutual information between (p,q,r) and y (corresponding to the term $I(q^{k},r^{k},p^{k}\to y^{k}\|x^{k})$) can be thought of as being “leaked” through s, thus bypassing the forward link from x to y. This provides an intuitive interpretation for (5).

  **Remark 1.***Theorem 1 implies that $I(x^{k}\to y^{k})$ is only a part of (or at most equal to) the information “flow” between all the exogenous signals entering the loop outside the link x→y (namely (q,r,p)), and y. In particular, if (p,q,r) were deterministic, then $I(x^{k}\to y^{k})=0$, regardless of the blocks $S_{1},\ldots ,S_{4}$ and irrespective of the nature of s.*
2. Our second main result is the following theorem, which relates directed information involving four different sequences internal to the loop. The proof is in Appendix A on page 19.
  **Theorem** **2** (Full Closed-Loop Directed Data-Processing Inequality)**.** *Consider the system shown in Figure 1a.*
  *(a)*  *If (q,s)⫫(r,p) and q⫫s, or if (p,s)⫫(r,q) and p⫫s, then:*
    (6) $$\begin{array}{c} I\left(x^{k}\to y^{k}\right)\geq I\left(e^{k}\to u^{k}\right). \end{array}$$
  *(b)*  *If (q,s)⫫(r,p) and $q_{i+1}^{k}↔q^{i}↔s^{i}$ for i=1,2,…,k−1, then:*
    (7) $$\begin{array}{c} I\left(x^{k}\to y^{k}∥q^{k}\right)\geq I\left(e^{k}\to u^{k}\right). \end{array}$$
  (The Markov chain notation t↔v↔w means “t and w are independent when v is given”.) To the best of our knowledge, Theorem 2 is the first result available in the literature providing a lower bound to the gap between two instances of nested directed information, involving four different signals inside the feedback loop. This result can be seen as the first full extension of the open-loop (traditional) data-processing inequality, to arbitrary closed-loop scenarios. (Notice that there is no need to consider systems with more than four mappings, since all external signals entering the loop between a given pair of internal signals can be regarded as exogenous inputs to a single equivalent deterministic mapping.)
3. Our third main contribution is introducing the notion of *in-the-loop* (ITL) transmission rate (in Section 6) for the (seldom considered) channel-coding scenario in which the messages to be transmitted and the communication channel are internal to a feedback loop. We show that the supremum of the directed information rate across such a channel upper bounds the achievable ITL transmission rates. Moreover, we present an example in which this upper bound is attainable. This gives further operational meaning to the directed information rate in closed-loop scenarios.
4. Finally, we provide additional examples of the applicability of our results by discussing how they allow one to obtain the generalizations of two fundamental inequalities known in networked control literature. The first one appears in [18] (Lemma 4.1) and is written in (12) below. This generalization is a consequence of Theorem 4 and is discussed in Remarks 3 and 5 below. The second generalization applies to [20] (Theorem 4.1) and is described on page 6 below. It is an application of Theorem 2 that has just been carried out by the authors in [26], which is all the more important since, as we also reveal in that note, there is a flaw in the proof of [20] (Theorem 4.1).

A key ingredient in proving most of our theorems is provided by Lemma 1, stated in Section 2.5. It allows one to rigorously establish some of the non-trivial conditional independencies that arise in a feedback loop with several (possibly stochastic) dynamic systems.

Put together, the law of conservation of information flow from Theorem 1, our extension of the data processing inequality to general feedback systems from Theorem 2, and our other results constitute both a conceptual framework and a toolbox for addressing information flow problems in feedback systems. We are convinced that this contribution will be instrumental in establishing new results on, e.g., rate-distortion and channel capacity problems with feedback.

The literature review presented next will allow the reader to further assess the novelty and relevance of our results.

### 1.2. Existing Related Results

There exist several results characterizing the relationship between $I(x^{k}\to y^{k})$ and $I(x^{k};y^{k})$. First, it is well known that $I\left(x^{k}\to y^{k}\right)\leq I\left(x^{k};y^{k}\right)$, with equality if and only if $y^{k}$ is causally related to $x^{k}$ [13]. A conservation law of mutual and directed information has been found in [27], which asserts that $I\left(x^{k}\to y^{k}\right)+I\left(0*y^{k-1}\to x^{k}\right)=I\left(x^{k};y^{k}\right)$, where $0*y^{k-1}$ denotes the concatenation $0,y\left(1\right),\ldots ,y^{k-1}$.

Given its prominence in settings involving feedback, it is perhaps in these scenarios where the directed information becomes most important. For instance, the directed information has been instrumental in characterizing the capacity of channels with feedback (see, e.g., [15,16,28] and the references therein), as well as the rate-distortion function in setups involving feedback [5,20,21,22,29].

For the simple case in which all the systems ${\left\{S_{i}\right\}}_{i=1}^{4}$ are linear time invariant (LTI) and stable, and assuming p,x,q=0 (deterministically), it was shown in [30] that $I(r^{k}\to e^{k})$ does not depend on whether there is feedback from e to u or not.

Inequalities between mutual and directed information in a less restricted setup, shown in Figure 1b, have been found in [18,19]. In that setting (a networked-control system), *G* is a strictly causal LTI dynamic system having (vector) state sequence ${\left\{x\left(i\right)\right\}}_{i=0}^{\infty }$, with p≜x(0) being the random initial state in its state-space representation. The external signal r (which could correspond to a disturbance) is statistically independent of s, the latter corresponding to, for example, side information or channel noise. Both are also statistically independent of p.

The blocks labeled *E*, *D*, and *f* correspond to an encoder, a decoder, and a channel, respectively, all of which are causal. The channel *f* maps $s^{k}$ and $x^{k}$ to y(k) in a possibly time-varying manner, i.e., $y\left(k\right)=f\left(k,x^{k},s^{k}\right).$ Similarly, the concatenation of the encoder, the channel and the decoder, maps $s^{k}$ and $w^{k}$ to u(k) as a possibly time-dependent function $u\left(k\right)=\psi \left(k,w^{k},s^{k}\right).$ Under these assumptions, the following fundamental result was shown in [19] (Lemma 5.1):

(8) $$\begin{array}{c} I\left(r^{k},p\;;\;u^{k}\right)\geq I\left(r^{k};u^{k}\right)+I\left(p;e^{k}\right). \end{array}$$

By further assuming in [19] that the decoder *D* in Figure 1b is deterministic, the following Markov chain naturally holds,

(9) $$\begin{array}{c} \left(p,r^{k}\right)⟷y^{k}⟷u^{k}, \end{array}$$

leading directly to:

(10) $$\begin{array}{c} I\left(r^{k},p\;;\;y^{k}\right)\geq I\left(r^{k};u^{k}\right)+I\left(p;e^{k}\right), \end{array}$$

which is found in the proof of [19] (Corollary 5.3). The deterministic nature of the decoder *D* played a crucial role in the proof of this result, since otherwise the Markov chain (9) does not hold, in general, due to the feedback from u to y.

Notice that both (8) and (10) provide lower bounds to mutual information as the sum of two mutual information terms, each of them relating a signal *external* to the loop (such as $p,r^{k}$) to a signal *internal* to the loop (such as $u^{k}$ or $y^{k}$). Instead, the inequality:

(11) $$\begin{array}{c} I\left(x^{k}\to y^{k}\right)\geq I\left(r^{k};y^{k}\right), \end{array}$$

which holds for the system in Figure 1a and appears in [13] (Theorem 3) (and rediscovered later in [17] (Lemma 4.8.1)), involves the directed information between two internal signals and the mutual information between the second of these and an external sequence.

**Remark** **2.** *By using* (22), *$I\left(p^{k},q^{k},r^{k};y^{k}\right)=I\left(r^{k};y^{k}\right)+I\left(p^{k},q^{k};y^{k}|r^{k}\right)$. Then, applying Theorem 1, we recover* (11), *whenever s⫫(q,r,p). Thus, [13,17] (Theorem 3) and (Lemma 4.8.1)) can be obtained as a corollary of Theorem 1.*

A related bound, similar to (10) but involving information rates and with the leftmost mutual information replaced by the directed information from $x^{k}$ to $y^{k}$ (which are two signals internal to the loop), has been obtained in [18] (Lemma 4.1) for the networked control system of Figure 1b:

(12) $$\begin{array}{c} \bar{I}\left(x\to y\right)\geq \bar{I}\left(r;u\right)+\lim_{k\to \infty }\frac{I(p;e^{k})}{k}, \end{array}$$

with $\bar{I}\left(x\to y\right)≜\lim_{k\to \infty }\frac{1}{k}I\left(x^{k}\to y^{k}\right)$ and $\bar{I}\left(r;u\right)≜\lim_{k\to \infty }\frac{1}{k}I\left(r^{k};u^{k}\right)$, provided $\sup_{i\geq 0}E\;\left[x{\left(i\right)}^{T}x\left(i\right)\right]<\infty$. This result relies on three assumptions: (a) that the channel *f* is memory-less and satisfies a “conditional invertibility” property, (b) a finite-memory condition, and (c) a fading-memory condition, the latter two related to the decoder *D* (see Figure 1).

It is worth noting that, as defined in [18], these assumptions upon *D* exclude the use of side information by the decoder and/or the possibility of *D* being affected by random noise or having a random internal state that is non-observable (please see [18] for a detailed description of these assumptions).

**Remark** **3.** *In Section 4 we present Theorem 4, which yields* (12) *as a special case, but for the general system of Figure 1a and with no other assumption than mutual independence between r,p,q,s. Moreover, since with this independence condition Theorem 1 yields $I\left(x^{k}\to u^{k}\right)=I\left(r^{k},p^{k};u^{k}\right)$, the same happens with* (8).

The inequality (11) has been extended in [16] (Theorem 1), for the case of discrete-valued random variables and assuming s⫫(r,p,q), as the following identity (written in terms of the signals and setup shown in Figure 1a):

(13) $$\begin{array}{c} I\left(x^{k}\to y^{k}\right)=I\left(p^{k},y^{k}\right)+I\left(x^{k}\to y^{k}|p^{k}\right). \end{array}$$

Letting q=s in Figure 1a and with the additional assumption that (p,s)⫫q, it was also shown in [16] (Theorem 1) that:

(14) $$\begin{array}{c} I\left(x^{k}\to y^{k}\right)=I\left(p^{k};y^{k}\right)+I\left(q^{k-1};y^{k}\right)+I\left(p^{k};q^{k-1}|y^{k}\right), \end{array}$$

for the cases in which u(i)=y(i)+q(i) (i.e., when the concatenation of $S_{4}$ and $S_{1}$ corresponds to a summing node). In [16], (13) and (14) play important roles in characterizing the capacity of channels with noisy feedback.

To the best of our knowledge, (8), (10), (11)–(14) are the only results available in the literature that lower bound the difference between internal-to-internal directed information and external-to-internal mutual information. There exist even fewer published results in relation to inequalities between two directed information terms involving only signals internal to the loop. To the best of our knowledge, the only inequality of this type in the literature is the one found in the proof of Theorem 4.1 of [20]. The latter takes the form of a (conditional) data-processing inequality for directed information in closed-loop systems, and states that:

(15) $$\begin{array}{c} I\left(x^{k}\to y^{k}\|q^{k}\right)\geq I\left(x^{k}\to u^{k}\right), \end{array}$$

provided: q⫫(r,p) and if $S_{4}$ is such that $y^{i}$ is a function of $\left(u^{i},q^{i}\right)$ (i.e., if $S_{4}$ is conditionally invertible) ∀i.

**Figure 3:**
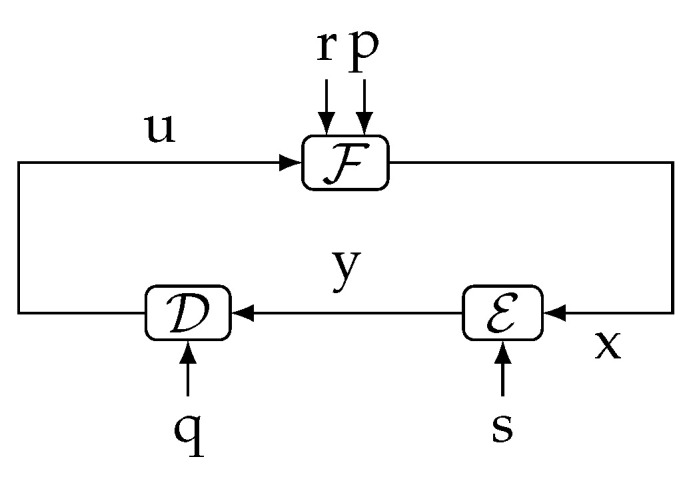
The networked control system considered in [20] (Figure 2), slightly simplified. The variables r,p,x,y correspond to $d,x_{o},y,s$ in [20], respectively.

**Figure 4:**
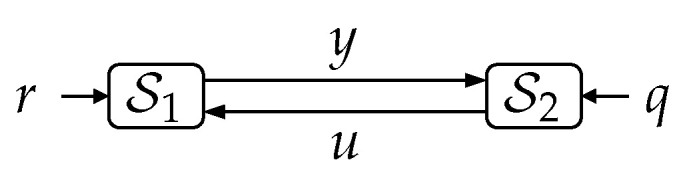
Two arbitrary causal systems $S_{1},S_{2}$ interconnected in a feedback loop. The exogenous signals r,q are mutually independent.

Inequality (15) plays a crucial role in [20], since it allows [20] (Thm. 4.1) to lower bound the average data rate across a digital error-free channel by a directed information. The setup considered in that theorem is shown in Figure 3, where F is a plant, and E, D are the (source) encoder and decoder, respectively. In this figure, the variables that have been adapted to match those in Figure 4a (r,p,x correspond to disturbance, initial state, and plant output, respectively). Assuming (r,p)⫫(q,s) and a conditionally invertible decoder, and letting R(i) be the expected length (in bits) necessary for a binary representation of y(i) given $q^{i}$, it states that $\frac{1}{k}\sum_{i=1}^{k}R\left(i\right)\geq \frac{1}{k}I\left(x^{k}\to u^{k}\right),\;k=1,2,\ldots .$ This is a key result, because, combined with [20] (Equation (9)), it yields:

(16) $$\begin{array}{c} \frac{1}{k}I\left(x^{k}\to u^{k}\right)\leq \frac{1}{k}\sum_{i=1}^{k}R\left(i\right)\leq \frac{1}{k}I\left(x^{k}\to u^{k}\right)+1\;\;\;\;\left[\mathrm{bits}/\mathrm{sample}\right],\;\;\;\;k=1,2,\ldots . \end{array}$$

This result highlights the operational meaning of the directed information as a lower bound (tight to within one bit) to the data rate of any given source code in a closed-loop system. This fact has been a crucial ingredient in characterizing the best rate performance achievable in Gaussian linear quadratic networked control [23,31], demonstrating the relevance of directed data-processing inequalities.

Unfortunately, as we will reveal in [26], the proof of [20] (Theorem 4.1) turns out to be invalid, since it relies upon [20] (Lemma 4.2), whose first claim does not hold. In [26] we use Theorem 2 to prove Theorem 4.1 of [20] without requiring a conditionally invertible decoder. This further illustrates the applicability of our results.

In [23] (Lemma 1) another data-processing inequality is stated, which for the system in Figure 1a is equivalent to:

(17) $$\begin{array}{c} I\left(x^{k}\to y^{k}∥u_{+}^{k-1}\right)\geq I\left(x^{k}\to u^{k}\right),\;\;\;\;k=1,2,\ldots \end{array}$$

where: $I\left(x^{k}\to y^{k}∥u_{+}^{k-1}\right)≜\sum_{i=1}^{k}I\left(x^{i};y\left(i\right)|y^{i-1},u^{i-1}\right)$. However, in [23] the blocks $S_{3},\;S_{4}$ are defined implicitly, writing instead their input–output relation as collections of stochastic kernels $P(y\left(i\right)|x^{i},y^{i-1})$, $P(u\left(i\right)|y^{i},u^{i-1})$, i=1,2,…. The notation P(t|v,w) is to be understood as the conditional distribution of t given (v,w). Crucially, this entails the implicit assumption that given $x^{i}$ and $y^{i-1}$, y(i) is independent of every other signal in the system (and likewise for $P(u\left(i\right)|y^{i},u^{i-1})$). In the representation of Figure 1a, this corresponds to assuming q⫫s and (q,s)⫫(r,p).

**Remark** **4.** *The conditioning on the side information q in both Theorem 2 and [20] (Theorem 4.1) is motivated by the use of entropy coded subtractively dithered quantization (ECSDQ) in obtaining the upper bound in* (16). *For such a scenario, the sequences q and s are identical and correspond to the dither signal, which is independent of r,p. This satisfies the requirements of* (6) *in Theorem 2 and of [20] (Theorem 4.1), but not the assumption that q⫫s and (q,s)⫫(r,p) implicit in [23] (Lemma 1), which yields* (17). *In spite of this, Lemma 1 of [23] is used in that paper to prove the lower bound in [23] (Equation (8)), an analogue of* (16) *which also considers the use of ECSDQ for the rate term and its upper bound.*

### 1.3. Outline of the Paper

The remainder of the paper continues with some preliminary definitions and results in Section 2, the last of which is Lemma 2, a crucial tool for proving most of our theorems. Then follows the proof of Theorem 1 in Section 3. Section 4 presents additional inequalities relating mutual information between external–internal signal pairs, and directed information from one internal signal to another internal signal. These results can be seen as extensions or consequences of Theorem 1. Then we develop in Section 5 inequalities between two nested directed information expressions. Such results are the precursors of Theorem 2 and, as such, play a key role in its proof (which opens Appendix A). The notions and results associated with in-the-loop channel coding are developed in Section 6. The main conclusions of this work are presented in Section 7. Appendix A provides the proofs that are not written right after their corresponding theorems.

An earlier version of this work was made publicly available on arxiv.org [32] and, as such, it was cited in [23,31,33,34,35].

## 2. Preliminaries

### 2.1. Notation

The set of natural numbers is denoted N. Random variables are denoted using non-italic characters, such as x. We write $x^{i}$ to represent the sequence x(1),x(2),…,x(i). We write x⫫y to express that x and y are independent. We use Pr{“outcome”} to denote the probability of a specific outcome of one or more random variables. For example, Pr{x=x,y∈Y} is the probability that x=1 and y is in a given set Y. Likewise, Pr{“outcome”|“outcome 2”} is the conditional probability of “outcome 1” given “outcome 2”. The Markov-chain notation x↔y↔z means Pr{x∈X,z∈Z|y∈Y}=Pr{x∈X|y∈Y}Pr{z∈Z|y∈Y}, for every choice of the sets X,Y,Z in the event spaces of x,y, and z, respectively. For two probability measures μ,ν on a common event space U the notation μ≪ν means that μ is absolutely continuous with respect to ν, i.e., that ∀U∈U:ν(U)=0⇒μ(U)=0.

### 2.2. Mutual Information

Let (Ω,F,P) be a probability space, and $\left(X,F_{X}\right)$ and $\left(Y,F_{Y}\right)$ be measurable spaces, and consider the random variables x:Ω→X, y:Ω→Y. Define $M≜F_{X}⊗F_{Y}$, i.e, the σ-algebra generated by the rectangles {A×B:A∈X,B∈Y}. Consider a probability space (X×Y,M,m) where *m* is the (joint) distribution of (x,y), i.e., $m=P\circ {\left(x,y\right)}^{-1}$.

Denote the marginal probability distributions of x and y by μ and ν, respectively, where:

(18) $$\begin{array}{cc} \mu (A) & =m\left(A\times Y\right),\;\;\;\;A\in F_{X} \end{array}$$

(19) $$\begin{array}{cc} \nu (B) & =m\left(X\times B\right),\;\;\;\;B\in F_{Y} \end{array}$$

Define the product measure π≜μ×ν on (X×Y,M).

**Definition** **1.** *With the above definitions, the mutual information between* x *and* y *is defined as:*

(20) $$\begin{array}{c} I\left(x;y\right)≜\int \log\left(\frac{dm}{d\pi }\right)dm, \end{array}$$

*where $\frac{dm}{d\pi }$ is the Radon–Nikodym derivative of m with respect to π [36].*

An ensemble of random variables has a standard alphabet if it takes values from a set A in a standard measurable space $\left(A,F_{A}\right)$ [37] (Section 1.4) and its probability measure is defined on $F_{A}$. For our purposes, it suffices to say that standard alphabets include discrete spaces, the real line, Euclidean vector spaces, and Polish spaces (i.e., complete separable metric spaces) [38].

**Lemma** **1** (Chain Rule of Mutual Information from [37] (Corollary 7.14))**.** *Suppose x,y,z are random variables with standard alphabets and with joint distribution $P_{xyz}$. Suppose also that there exists a product distribution $M_{xyz}=M_{x}\times M_{yz}$ such that $M_{xyz}\gg P_{xyz}$. (This is true, for example, if $P_{x}\times P_{yz}\gg P_{xyz}$.) Then:*

(21) $$\begin{array}{c} I(x;y,z)=I(x;y)+I(x;z|y). \end{array}$$

From [37] (Lemma 7.4 and Equation 7.28), we have that $I\left(x;y,z\right)<\infty \Rightarrow P_{x}\times P_{yz}\gg P_{xyz}$, and thus (21) also holds if I(x;y,z) is finite.

The conditional version of the chain rule of mutual information [39] (see also [37] (Corollary 2.5.1)) will be extensively utilized in the proofs of our results:

(22) $$\begin{array}{c} I(t,v;w|z)=I(v;w|z)+I(t;w|v,z). \end{array}$$

For discrete random variables x,y, taking values from the sets X,Y, respectively, the entropy of x is defined as:

(23) $$\begin{array}{c} H\left(x\right)≜-\sum_{x\in X}\mathrm{Pr}\left\{x=x\right\}\log\left(\mathrm{Pr}\{x=x\}\right) \end{array}$$

and the conditional entropy of x given y is defined as:

(24) $$\begin{array}{c} H\left(x|y\right)≜-\sum_{x\in X,y\in Y}\mathrm{Pr}\left\{x=x,y=y\right\}\log\left(\mathrm{Pr}\left\{x=x|y=y\right\}\right) \end{array}$$

The entropy satisfies the chain rule:

(25) $$\begin{array}{c} H(x,y)=H(x)+H(y|x)=H(y)+H(x|y) \end{array}$$

and is related to the mutual information as:

(26) $$\begin{array}{c} I(x;y)=H(x)+H(y)-H(x,y)=H(x)-H(x|y)=H(y)-H(y|x). \end{array}$$

### 2.3. System Description

We begin by providing a formal description of the systems labeled $S_{1}\ldots S_{4}$ in Figure 1a. Their input–output relationships are given by the possibly-varying deterministic mappings (For notational simplicity, we omit writing their time dependency explicitly):

(27a) $$\begin{array}{cc} e(i) & =S_{1}\left(u^{i-d_{1}\left(i\right)},r^{i}\right), \end{array}$$

(27b) $$\begin{array}{cc} x(i) & =S_{2}\left(e^{i-d_{2}\left(i\right)},p^{i}\right), \end{array}$$

(27c) $$\begin{array}{cc} y(i) & =S_{3}\left(x^{i-d_{3}\left(i\right)},s^{i}\right), \end{array}$$

(27d) $$\begin{array}{cc} u(i) & =S_{4}\left(y^{i-d_{4}\left(i\right)},q^{i}\right), \end{array}$$

where r,p,s,q are exogenous random signals and the (possibly time-varying) delays $d_{1},d_{2},d_{3},d_{4}\in \left\{0,1,\ldots \right\}$ are such that:

$$d_{1}\left(k\right)+d_{2}\left(k\right)+d_{3}\left(k\right)+d_{4}\left(k\right)\geq 1,\;\;\;\;\forall k\in N.$$

That is, the concatenation of $S_{1},\ldots ,S_{4}$ has a delay of at least one sample. For every i∈{1,…,k}, $r\left(i\right)\in R^{n_{r}\left(i\right)}$, i.e., r(i) is a real random vector whose dimension is given by some function $n_{r}:\left\{1,\ldots ,k\right\}\to N$. The other sequences (q,p,s,x,y,u) are defined likewise.

### 2.4. A Necessary Modification of the Definition of Directed Information

As stated in [13], the directed information (as defined in (3)) is a more meaningful measure of the flow of information between $x^{k}$ and $y^{k}$ than the conventional mutual information $I\left(x^{k};y^{k}\right)=\sum_{i=1}^{k}I\left(y\left(i\right);x^{k}|y^{i-1}\right)$ when there exists causal feedback from y to x. In particular, if $x^{k}$ and $y^{k}$ are discrete-valued sequences, the input and output, respectively, of a forward channel, and if there exists *strictly causal* perfect feedback, so that x(i)=y(i−1) (a scenario utilized in [13] as part of an argument in favor of the directed information), then the mutual information becomes:

$$\begin{array}{cc} I(x^{k};y^{k})\; & =H\left(y^{k}\right)-H\left(y^{k}|x^{k}\right)=H\left(y^{k}\right)-H\left(y^{k}|y^{k-1}\right)=H\left(y^{k}\right)-H\left(y\left(k\right)|y^{k-1}\right)\\ & =H(y^{k-1}). \end{array}$$

Thus, when strictly causal feedback is present, $I(x^{k};y^{k})$ fails to account for how much information about $x^{k}$ has been conveyed to $y^{k}$ through the forward channel that lies between them.

It is important to note that in [13] (as well as in many works concerned with communications), the forward channel is instantaneous, i.e., it has no delay. Therefore, if a feedback channel is utilized, then this feedback channel must have a delay of at least one sample, as in the example above. However, when studying the system in Figure 1a, we may need to evaluate the directed information between signals $x^{k}$ and $y^{k}$ which are, respectively, the input and output of a *strictly casual* forward channel (i.e., with a delay of at least one sample), whose output is instantaneously fed back to its input. In such a case, if one further assumes perfect feedback and sets x(i)=y(i), then, in the same spirit as before,

$$\begin{array}{cc} I(x^{k}\to y^{k})\; & =\sum_{i=1}^{k}I\left(y\left(i\right);x^{i}|y^{i-1}\right)=\sum_{i=1}^{k}\left[H\left(y\left(i\right)|y^{i-1}\right)-H\left(y\left(i\right)|x^{i},y^{i-1}\right)\right]=H\left(y^{k}\right). \end{array}$$

As one can see, Massey’s definition of directed information ceases to be meaningful if instantaneous feedback is utilized.

It is natural to solve this problem by recalling that, in the latter example, the forward channel had a delay, say *d*, greater than one sample. Therefore, if we are interested in measuring how much of the information in y(i), not present in $y^{i-1}$, was conveyed from $x^{i}$ through the forward channel, we should look at the mutual information $I(y\left(i\right);x^{i-d}|y^{i-1})$, because only the input samples $x^{i-d}$ can have an influence on y(i). For this reason, we introduce the following, modified notion of directed information.

**Definition** **2** (Directed Information with Forward Delay)**.** *In this paper, the directed information from $x^{k}$ to $y^{k}$ through a forward channel with a non-negative time varying delay of $d_{xy}\left(i\right)$ samples is defined as:*

(28) $$\begin{array}{c} I\left(x^{k}\to y^{k}\right)≜\sum_{i=1}^{k}I\left(y\left(i\right);x^{i-d_{xy}\left(i\right)}|y^{i-1}\right). \end{array}$$

For a zero-delay forward channel, the latter definition coincides with Massey’s [13].

Likewise, we adapt the definition of causally-conditioned directed information to the definition:

$$\begin{array}{c} I\left(x^{k}\to y^{k}\|e^{k}\right)≜\sum_{i=1}^{k}I\left(y\left(i\right);x^{i-d_{xy}\left(i\right)}|y^{i-1},e^{i}\right). \end{array}$$

where, as before, $d_{xy}\left(i\right)$ is the delay from x to y(i).

### 2.5. A Fundamental Lemma

The following result is an essential ingredient in the proof of most of our theorems:

**Lemma** **2.** *In the system shown in Figure 4, the exogenous signals r,q are mutually independent and $S_{1},S_{2}$ are deterministic (possibly time-varying) causal measurable functions characterized by $y^{i}=S_{1}\left(r^{i},u^{i}\right)$, $u^{i}=S_{2}\left(q^{i},y^{i-1}\right)$, ∀i∈{1,…}, with $y_{0}=y_{0}$ (deterministic). For this system, and for every 0≤j≤i≤k such that i−j≤1 and i≥1, the following Markov chain holds:*

(29) $$\begin{array}{c} r^{k}⟷\left(u^{i},y^{j}\right)⟷q^{k},\;\;\;\;\forall k\in N. \end{array}$$

**Proof.**  Let R,Q,U,Y be the event spaces of $r^{k},q^{k},u^{i},y^{j}$, respectively. Since $y^{j}=S_{1}\left(r^{j},u^{j}\right)$ and $u^{i}=S_{2}\left(q^{i},y^{i-1}\right)$ are deterministic measurable functions, it follows that for every possible pair of events U∈U, Y∈Y, the preimage sets $R_{U,Y}≜\left\{r^{k}:S_{1}\left(r^{j},u^{j}\right)\in Y,u^{i}\in U\right\}$ and $Q_{U,Y}≜\left\{q^{k}:S_{2}\left(q^{i},y^{i-1}\right)\in U,y^{j}\in Y\right\}$ are also deterministic and belong to R and Q, respectively. Thus, $\left(u^{i},y^{j}\right)\in U\times Y\Leftrightarrow \left(r^{k}\in R_{U,Y},q^{k}\in Q_{U,Y}\right)$. This means that for every pair of events R∈R,Q∈Q,

Pr{rk∈R,qk∈Q|yj∈Y,ui∈U}=(a)Pr{rk∈R,qk∈Q|rk∈RU,Y,qk∈QU,Y}=(b)Pr{rk∈R∩RU,Y,qk∈Q∩QU,Y}Pr{rk∈RU,Y,qk∈QU,Y}=(c)Pr{rk∈R∩RU,Y}Pr{rk∈RU,Y}·Pr{qk∈Q∩QU,Y}Pr{qk∈QU,Y}=(d)Pr{rk∈R∩RU,Y}Pr{qk∈QU,Y}Pr{rk∈RU,Y}Pr{qk∈QU,Y}·Pr{qk∈Q∩QU,Y}Pr{rk∈RU,Y}Pr{qk∈QU,Y}Pr{rk∈RU,Y}=(d)Pr{rk∈R∩RU,Y,qk∈QU,Y}Pr{rk∈RU,Y,qk∈QU,Y}·Pr{qk∈Q∩QU,Y,rk∈RU,Y}Pr{qk∈QU,Y,rk∈RU,Y}=(e)Pr{rk∈R|rk∈RU,Y,qk∈QU,Y}·Pr{qk∈Q|qkQU,Y,rk∈RU,Y}=(f)Pr{rk∈R|yj∈Y,ui∈U}·Pr{qk∈Q|yj∈Y,ui∈U}

where (a) and (f) follow because of the equivalence between the events $\left(y^{j}\in Y,u^{i}\in U\right)$ and $\left(r^{k}\in R_{U,Y},q^{k}\in Q_{U,Y}\right)$, (b) and (e) follow from Bayes rule, and (c) and (d) are true because $r^{k}⫫q^{k}$. This completes the proof. □

## 3. Proof of Theorem 1

**Figure 5:**
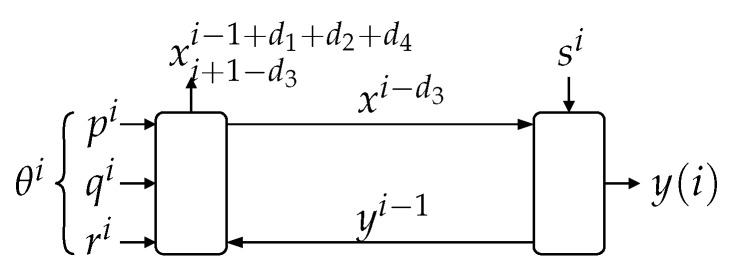
Representation of the system of Figure 1b highlighting the dependency between *p*, *q*, *r*, *s*, *x*, and *y*. The dependency on *i* of the delays $d_{1}\left(i\right),\ldots ,d_{4}\left(i\right)$ is omitted for clarity.

It is clear from Figure 1a and from (27) that the relationship between r, p, q, s, x, and y can be represented by the diagram shown in Figure 5.

From this diagram and Lemma 2 it follows that if s is independent of (r,p,q), then the following Markov chain holds:

(30) $$\begin{array}{cc} y(i) & ⟷\left(x^{i-d_{3}\left(i\right)},y^{i-1}\right)⟷\left(p^{i},q^{i},r^{i}\right). \end{array}$$

Denoting the triad of exogenous signals $p^{k},q^{k},r^{k}$ by:

(31) $$\begin{array}{c} \theta^{k}≜\left(p^{k},q^{k},r^{k}\right), \end{array}$$

we have the following:

$$\begin{array}{cc} I(x^{k}\to y^{i})\; & =\sum_{i=1}^{k}I\left(y\left(i\right);x^{i-d_{3}\left(i\right)}|y^{i-1}\right)\\ & \overset{\left(22\right)}{=}\sum_{i=1}^{k}\left[I\left(\theta^{i},x^{i-d_{3}\left(i\right)};y\left(i\right)|y^{i-1}\right)-I\left(\theta^{i};y\left(i\right)|x^{i-d_{3}\left(i\right)},y^{i-1}\right)\right] \end{array}$$

(32a) $$\;\overset{\left(a\right)}{=}\sum_{i=1}^{k}\left[I\left(\theta^{i},y\left(i\right)|y^{i-1}\right)-I\left(\theta^{i};y\left(i\right)|x^{i-d_{3}\left(i\right)},y^{i-1}\right)\right]$$

(32b) $$\;\overset{\left(b\right)}{\leq }\sum_{i=1}^{k}I\left(\theta^{i},y\left(i\right)|y^{i-1}\right)\overset{\left(c\right)}{\leq }\sum_{i=1}^{k}I\left(\theta^{k},y\left(i\right)|y^{i-1}\right)$$

(32c) $$=I\left(\theta^{k};y^{k}\right)\;$$

In the above, (a) follows from the fact that, if $y^{i-1}$ is known, and then $x^{i-d_{3}\left(i\right)}$ is a deterministic function of $\theta^{i}$. The resulting sums on the right-hand side of (32a) correspond to $I\left(q^{k},r^{k},p^{k}\to y^{k}\right)-I\left(q^{k},r^{k},p^{k}\to y^{k}\|x^{k}\right)$, thereby proving the first part of the theorem, i.e., the equality in (5). In turn, (b) stems from the non-negativity of mutual information turn into equality if s⫫(r,p,q), as a direct consequence of the Markov chain in (30). Finally, equality holds in (c) if s⫫(q,r,p), since y depends causally upon θ. This shows that equality in (5) is achieved if s⫫(q,r,p), completing the proof.

## 4. Relationships between Mutual and Directed Information

The following result provides an inequality relating $I(x^{k}\to y^{k})$ with the separate flows of information $I(r^{k};y^{k})$ and $I(p^{k},q^{k}\;;\;y^{k})$.

**Theorem** **3.** *For the system shown in Figure 1a, if s⫫(p,q,r) and $r^{k}⫫\left(p^{k},q^{k}\right)$, then:*

(33) $$\begin{array}{cc} I(x^{k}\to y^{k})\; & \geq I\left(r^{k};y^{k}\right)+I\left(p^{k},q^{k}\;;\;y^{k}\right). \end{array}$$

*with equality if and only if the Markov chain $\left(p^{k},q^{k}\right)↔y^{k}↔r^{k}$ holds.*

Theorem 3 shows that, provided (p,q,r)⫫s, $I(x^{k}\to y^{k})$ is lower bounded by the sum of the individual flows from all the subsets in any given partition of $\left(p^{k},q^{k},r^{k}\right)$, to $y^{k}$, provided these subsets are mutually independent. Indeed, both Theorems 1 and 3 can be generalized for any appropriate choice of external and internal signals. More precisely, let Θ be the set of all external signals in a feedback system. Let α and β be two internal signals in the loop. Define $\Theta_{\alpha ,\beta }\subset \Theta$ as the set of exogenous signals that are introduced to the loop at every subsystem $S_{i}$ that lies in the path going from α to β. Thus, for any $\rho \in \Theta \backslash \Theta_{\alpha ,\beta }$, if $\Theta_{\alpha ,\beta }⫫\Theta \backslash \Theta_{\alpha ,\beta }$, we have that (5) and (32) become:

(34) $$\begin{array}{cc} I(\alpha \to \beta ) & =I(\Theta \backslash \left\{\Theta_{\alpha ,\beta }\right\};\beta ), \end{array}$$

(35) $$\begin{array}{cc} I(\alpha \to \beta )-I(\rho ;\beta ) & \geq I(\Theta \backslash \left\{\rho \cup \Theta_{\alpha ,\beta }\right\};\beta ), \end{array}$$

respectively.

To finish this section, we present a stronger, non-asymptotic version of inequality (12):

**Theorem** **4.** *In the system shown in Figure 1a, if (r,p,q,s) are mutually independent, then:*

(36) $$\begin{array}{cc} I(x^{k}\to y^{k})\; & =I\left(r^{k};u^{k}\right)+I\left(p^{k};e^{k}\right)+I\left(q^{k};y^{k}\right)+I\left(p^{k};u^{k}|e^{k}\right)+I\left(r^{k},p^{k};y^{k}|u^{k}\right). \end{array}$$

**Remark** **5.** *As anticipated, Theorem 4 can be seen as an extension of* (12) *to the more general setup shown in Figure 1a, where the assumptions made in [18] (Lemma 4.1) do not need to hold. In particular, letting the decoder D and p in Figure 1b correspond to $S_{4}$ and $p^{k}$ in Figure 1a, respectively, we see that inequality* (12) *holds even if the channel f has memory or D and E have independent initial states, or if the internal state of D is not observable [40].*

**Figure 6:**
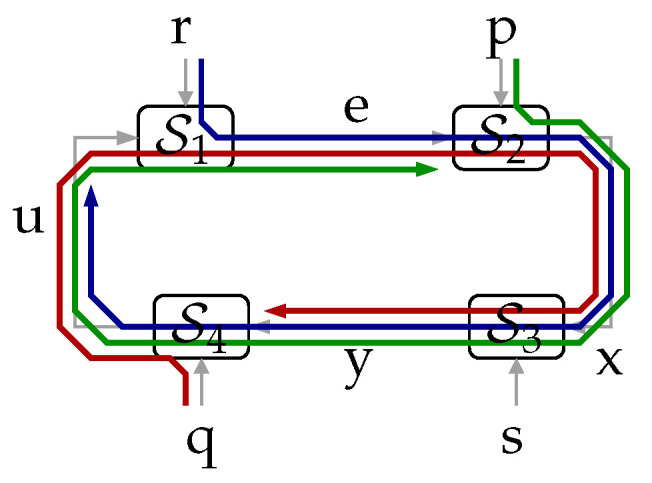
A representation of the three first information flows on the right-hand side of (36).

Theorem 4 also admits an interpretation in terms of information flows. This can be appreciated in the diagram shown in Figure 6, which depicts the individual full-turn flows (around the entire feedback loop) stemming from q, r, and p. Theorem 4 states that the sum of these individual flows is a lower bound for the directed information from x to y, provided q,r,p,s are independent.

## 5. Relationships between Nested Directed Information

This section presents three closed-loop versions of the data-processing inequality *relating two directed information terms*, both between pairs of signals *internal* to the loop. As already mentioned in Section 1, to the best of our knowledge, the first inequality of this type to appear in the literature is the one in Theorem 4.1 in [20] (see (15)). Recall that the latter result stated that $I\left(x^{k}\to y^{k}\|q^{k}\right)\geq I\left(x^{k}\to u^{k}\right)$, requiring $S_{4}$ to be such that $y^{i}$ is a deterministic function of $\left(u^{i},q^{i}\right)$ and that q⫫(r,p). The following result presents another inequality that also relates two nested directed information terms, namely, $I(x^{k}\to y^{k})$ and $I(e^{k}\to y^{k})$, but requiring only that s⫫(q,r,p).

**Theorem** **5.** *For the closed-loop system in Figure 1b, if (q,r,p)⫫s, then:*

(37) $$\begin{array}{cc} I(x^{k}\to y^{k}) & \geq I(e^{k}\to y^{k}). \end{array}$$

Notice that Theorem 5 does not require p to be independent of r or q. This may seem counterintuitive upon noting that p enters the loop between the link from e to x.

The following theorem is an identity between two directed information terms involving only internal signals. It can also be seen as a complement to Theorem 5, since it can be directly applied to establish the relationship between $I(e^{k}\to y^{k})$ and $I(e^{k}\to u^{k})$.

**Theorem** **6.** *For the system shown in Figure 1a, if s⫫(q,r,p), then:*

(38) $$\begin{array}{c} I\left(x^{k}\to y^{k}\right)\geq I\left(x^{k}\to u^{k}\right)+I\left(q^{k}\;;\;y^{k}\right)+I\left(r^{k},p^{k};y^{k}|u^{k}\right)+I\left(q^{k};r^{k}|u^{k},y^{k}\right), \end{array}$$

*with equality if, in addition, q⫫(r,p). In the latter case, it holds that:*

(39) $$\begin{array}{c} I\left(x^{k}\to y^{k}\right)=I\left(x^{k}\to u^{k}\right)+I\left(q^{k}\;;\;y^{k}\right)+I\left(r^{k},p^{k};y^{k}|u^{k}\right). \end{array}$$

Notice that by requiring additional independence conditions upon the exogenous signals (specifically, q⫫s), Theorem 6 (and, in particular, (39)) yields:

(40) $$\begin{array}{c} I\left(x^{k}\to y^{k}\right)\geq I\left(x^{k}\to u^{k}\right), \end{array}$$

which strengthens the inequality in [20] (Theorem 4.1) (stated above in (15)). More precisely, (40) does not require conditioning one of the directed information terms and holds irrespective of the invertibility of the mappings in the loop.

## 6. Giving Operational Meaning to the Directed Information: In-the-Loop Channel Coding

In this section we introduce the notions of in-the-loop transmission rate and capacity and show that they are related by the directed information rate across the channel in the same feedback loop. This provides another example to illustrate the applicability of Theorems 1 and 2 and also provides further operational meaning to the directed information rate.

**Figure 7:**
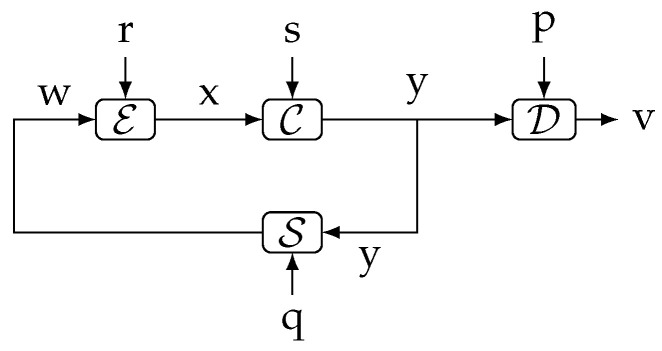
A communication feedback system in which the messages and the channel output are within the loop.

Consider the scheme shown in Figure 7, and suppose C is a noisy communication channel. Let E and D be the channel encoder and decoder, respectively, with r and p being side information sequences causally and independently available to each of them such that (r,p)⫫(s,q). This means that, for k=1,2,…,n,

(41) $$\begin{array}{cc} \left(p_{1}^{n},r_{k+1}^{n}\right) & ↔r_{1}^{k}↔\left(w_{1}^{k+1},x_{1}^{k},y_{0}^{k}\right). \end{array}$$

(42) $$\begin{array}{cc} p_{k+1}^{n} & ↔p_{1}^{k}↔r_{1}^{k} \end{array}$$

(43) $$\begin{array}{cc} p_{k+1}^{n} & ↔p_{1}^{k}↔\left(w_{1}^{k+1},x_{1}^{k},y_{0}^{k}\right). \end{array}$$

A crucial aspect of this scenario is the fact that the messages $w_{1}^{n},w_{n+1}^{2n},\ldots$ to be encoded are contained in the sequence w, a signal internal to the loop; they can be regarded as a corrupted version of the decoded messages, which comprise the sequence v. This is a key difference with respect to the available literature on feedback capacity, where, to the best of the authors’ knowledge, the messages are exogenous and the feedback signal only helps in the encoding task (exceptions can be found in some papers on networked control which consider in-the-loop channel coding, such as, e.g., [41,42]). In Figure 7, the latter standard scenario corresponds to encoding the sequence r.

The fact that the messages to be encoded bear information from the decoded message symbols requires one to redefine the notion of information transmission rate commonly used in the standard scenario. To see this, let w(k)∈W,k=1,2,…, for some finite alphabet W of cardinality $\left|W\right|$, and notice that the transmission rate definitions $\log(\left|W\right|)$ and $H(w_{1}^{n})/n$ are unsatisfactory if w(k)=y(k−1),k=1,2,…, i.e., if the messages to be transmitted are already available at the decoder (more generally, if there is no randomness in the feedback path). This suggests that a suitable notion of transmission rate for this scenario should exclude information that is already known by the receiver.

In view of the above, we propose the following notion of transmission rate for the case in which the messages to be transmitted are in the loop:

**Definition** **3.** *For the system described in Figure 1, the in-the-loop (ITL) transmission rate is defined as:*

(44) $$\begin{array}{c} R_{\mathrm{ITL}}^{n}≜\frac{1}{n}\sum_{k=1}^{n}H\left(w\left(k\right)|w_{1}^{k-1},y_{0}^{k-1},p_{1}^{k}\right). \end{array}$$

The meaning of the ITL transmission rate is further elucidated by considering the following scenarios:

1. If the feedback channel is deterministic, then w(k) is a deterministic function of $y_{0}^{k-1}$ and thus $R_{\mathrm{ITL}}^{n}=0$, as desired.
2. If the (forward) communication channel is noiseless, then at each time k−1, we have $y_{1}^{k-1}=w_{1}^{k-1}$. Therefore $R_{\mathrm{ITL}}^{n}=H\left(w_{1}^{n}|y_{0},p_{1}^{n}\right)/n$. Again, if the feedback channel is deterministic, the ITL transmission rate is zero.
3. In the absence of feedback, $R_{\mathrm{ITL}}^{n}=\frac{1}{n}H\left(w_{1}^{n}\right)$, recovering the notion of transmission rate of the case in which the messages are exogenous to the loop.

Thus, $R_{\mathrm{ITL}}^{n}$ can be interpreted as the sum of the information the encoder attempts to transmit at each sample time (expressed by the conditional entropy $H(w\left(k\right)|w_{1}^{k-1},y_{0}^{k-1},p_{1}^{k})$) that is novel for the transmitter (because of the conditioning on $w_{1}^{k-1}$) and novel for the receiver (due to the conditioning on $y_{0}^{k-1},p_{1}^{k}$).

**Theorem** **7.** *Consider the setup depicted in Figure 7, where E and D are the channel encoder and decoder, respectively, and C is the communication channel. Suppose the message and side-information samples w(k)∈W,r(k)∈R,k=1,2,…, respectively, where W and R are finite alphabets. Define the binary random variable $e_{n}$ to equal 1 if $v_{1}^{n}\neq w_{1}^{n}$ and 0 otherwise. Then, for every n∈N,*

(45) $$\begin{array}{c} R_{\mathrm{ITL}}^{n}\geq I\left(w_{1}^{n}\to y_{0}^{n}∥p_{1}^{n}\right), \end{array}$$

*with equality if and only if $H(w_{1}^{n}|y_{0}^{n},p_{1}^{n})=0$. Moreover,*

(46) $$\begin{array}{cc} \mathrm{Pr}\{e_{n}=1\}\; & =\frac{R_{\mathrm{ITL}}^{n}-\frac{1}{n}I\left(w_{1}^{n}\to y_{0}^{n}∥p_{1}^{n}\right)-\frac{1}{n}H\left(e_{n}|y_{0}^{n},p_{1}^{n}\right)}{\frac{1}{n}H\left(w_{1}^{n}|y_{0}^{n},p_{1}^{n},e_{n}=1\right)} \end{array}$$

(47) $$\begin{array}{cc} & \geq \frac{R_{\mathrm{ITL}}^{n}-\frac{1}{n}I\left(w_{1}^{n}\to y_{0}^{n}∥p_{1}^{n}\right)-1/n}{\log_{2}\left(\left|W\right|\right)} \end{array}$$

**Proof.**  Recall that:

(48) $$\begin{array}{cc} I(w_{1}^{n}\to y_{0}^{n}∥p_{1}^{n})\; & =\sum_{k=1}^{n}I\left(w_{1}^{k};y\left(k\right)|y_{0}^{k-1},p_{1}^{k}\right)=\sum_{k=1}^{n}H\left(w_{1}^{k}|y_{0}^{k-1},p_{1}^{k}\right)-\sum_{k=1}^{n}H\left(w_{1}^{k}|y_{0}^{k},p_{1}^{k}\right) \end{array}$$

On the other hand,

(49) $$\begin{array}{cc} nR_{\mathrm{ITL}}^{n} & =\sum_{k=1}^{n}H\left(w\left(k\right)|w_{1}^{k-1},y_{0}^{k-1},p_{1}^{k}\right)\overset{\left(\mathrm{cr}\right)}{=}\sum_{k=1}^{n}H\left(w_{1}^{k}|y_{0}^{k-1},p_{1}^{k}\right)-\sum_{k=2}^{n}H\left(w_{1}^{k-1}|y_{0}^{k-1},p_{1}^{k}\right) \end{array}$$

(50) $$\begin{array}{cc} & \;\overset{\left(48\right)}{=}\sum_{k=1}^{n}H\left(w_{1}^{k}|y_{0}^{k},p_{1}^{k}\right)-\sum_{k=2}^{n}H\left(w_{1}^{k-1}|y_{0}^{k-1},p_{1}^{k}\right)+I\left(w_{1}^{n}\to y_{0}^{n}∥p_{1}^{n}\right) \end{array}$$

(51) $$\begin{array}{cc} & \overset{\left(43\right)}{=}H\left(w_{1}^{k}|y_{0}^{k},p_{1}^{k}\right)+I(w_{1}^{n}\to y_{0}^{n}∥p_{1}^{n}),\; \end{array}$$

where the equality (cr) follows from the chain rule of entropy. This proves the first part of the theorem.

Let us now re-derive the first steps leading to Fano’s inequality, to include the side-information $p_{1}^{n}$ and to verify that it is not affected by the fact that w and y are within the loop.

(52) $$\begin{array}{cc} H(w_{1}^{n}|y_{0}^{n},p_{1}^{n})\; & \overset{\left(\mathrm{cr}\right)}{=}H\left(w_{1}^{n},e_{n}|y_{0}^{n},p_{1}^{n}\right)-H\left(e_{n}|y_{0}^{n},p_{1}^{n},w_{1}^{n}\right) \end{array}$$

(53) $$\begin{array}{cc} & \;\overset{\left(a\right)}{=}H\left(e_{n}|y_{0}^{n},p_{1}^{n}\right)+H\left(w_{1}^{n}|y_{0}^{n},p_{1}^{n},e_{n}\right) \end{array}$$

(54) $$\begin{array}{cc} & \;\overset{\left(b\right)}{=}H\left(e_{n}|y_{0}^{n},p_{1}^{n}\right)+H\left(w_{1}^{n}|y_{0}^{n},p_{1}^{n},e_{n}=1\right)\mathrm{Pr}\left\{e_{n}=1\right\}, \end{array}$$

where the equality (cr) follows from the chain rule of entropy and (a) holds because $H(e_{n}|y_{0}^{n},p_{1}^{n},w_{1}^{n})=0$ and from the chain rule, while (b) is because $H(w_{1}^{n}|y_{0}^{n},p_{1}^{n},e_{n}=0)=0$.

Substituting this into (51),

(55) $$\begin{array}{cc} nR_{\mathrm{ITL}}^{n} & =H\left(e_{n}|y_{0}^{n},p_{1}^{n}\right)+H\left(w_{1}^{n}|y_{0}^{n},p_{1}^{n},e_{n}=1\right)\mathrm{Pr}\left\{e_{n}=1\right\}+I\left(w_{1}^{n}\to y_{0}^{n}∥p_{1}^{n}\right). \end{array}$$

Noting that $H(e_{n}|y_{0}^{n},p_{1}^{n})\leq 1$ and $H\left(w_{1}^{n}|y_{0}^{n},p_{1}^{n},e_{n}=1\right)\leq n\log\left(\left|W\right|\right)$ leads directly to (46), comparing the proof. □

Theorem 7 allows one to draw an additional interpretation of the ITL transmission rate. We extend first the identity of [27] to include causal conditioning by $p_{1}^{n}$:

(56) $$\begin{array}{cc} I(w_{1}^{n};y_{0}^{n},p_{1}^{n})\; & =I\left(w_{1}^{n};y_{n}|y_{0}^{n-1},p_{1}^{n}\right)+I\left(w_{n};y_{0}^{n-1}|w_{1}^{n-1},p_{1}^{n}\right)+I\left(w_{1}^{n-1};y_{0}^{n-1},p_{1}^{n-1}\right) \end{array}$$

(57) $$\begin{array}{cc} & \;=\sum_{k=1}^{n}I\left(w_{1}^{k};y\left(k\right)|y_{0}^{k-1},p_{1}^{k}\right)+\sum_{k=1}^{n}I\left(w\left(k\right);y_{0}^{k-1}|w_{1}^{k-1},p_{1}^{k}\right)=I\left(w_{1}^{n}\to y_{0}^{n}∥p_{1}^{n}\right)+I\left(y_{0}^{n-1}\to w_{1}^{n}∥p_{1}^{n}\right), \end{array}$$

where:

(58) $$\begin{array}{c} I\left(y_{0}^{n-1}\to w_{1}^{n}∥p_{1}^{n}\right)≜\sum_{k=1}^{n}I\left(w\left(k\right);y_{0}^{k-1}|w_{1}^{k-1},p_{1}^{k}\right). \end{array}$$

It readily follows from (56) that:

(59) $$\begin{array}{c} H\left(w_{1}^{n}\right)-I\left(y_{0}^{n-1}\to w_{1}^{n}∥p_{1}^{n}\right)=I\left(w_{1}^{n}\to y_{0}^{n}∥p_{1}^{n}\right)+H\left(w_{1}^{n}|y_{0}^{n},p_{1}^{n}\right)\overset{\left(51\right)}{=}nR_{\mathrm{ITL}}^{n}. \end{array}$$

Thus, the ITL transmission rate corresponds to the entropy rate of the messages having extracted from it the information flowing from the decoder input to the messages.

The main result of this section is the following theorem, which asserts that the supremum of achievable ITL transmission rates is upper bounded by the directed information across the communication channel.

**Theorem** **8.** *Consider the setup depicted in Figure 7, where E and D are the channel encoder and decoder, respectively, and C is the communication channel. Then the supremum of achievable ITL transmission rates is upper bounded by the supremum of the directed information rate from *x* to *y* causally conditioned by $p_{1}^{n}$.*

**Proof.**  The result follows directly from Theorems 2 and 7. □

Thus, the supremum of $\lim_{n\to \infty }I\left(x_{1}^{n}\to y_{1}^{n}∥p_{1}^{n}\right)$ is an outer bound to the capacity region of ITL transmission rates.

In the following example, this bound is reachable.

**Figure 8:**
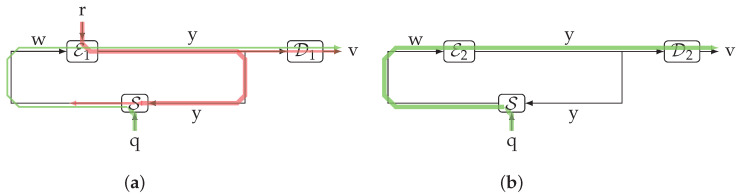
The feedback communication system considered in Example 1. In (**a**), encoder/decoder pair 1 yields a large mutual information between w and v by adding a backward information flow (in red) to the forward information flow (in green). The latter is only part of thee entropy rate of q. In (**b**), encoder/decoder pair 2 yields a smaller mutual information between w and v, but it corresponds to the greatest possible forward information flow, which coincides with the entropy rate of q. Thus, it is capacity achieving with respect to the ITL transmission rate.

**Example** **1.** *Consider the case in which the forward channel C in Figure 7 is transparent, i.e., y(k)=x(k) for k=0,1,…, as shown in Figure 8. Let y(k)∈{0,1,2,3}, k=0,1,…. Let q(0)=1 (deterministically) and q(1),q(2),… be binary and i.i.d. with Pr{q(k)=1}=α=0.9. The feedback channel S is defined by the following recursion:*

(60) $$\begin{array}{cc} w(k) & =\left\{\begin{array}{cc} q(k) & ,\;\;\mathrm{if}\;q(k-1)=(y(k-1)\;\mathrm{mod}\;2)\\ \left(y(k-1)\;\mathrm{mod}\;2\right) & \;,\;\mathrm{if}\;q(k-1)\neq (y(k-1)\;\mathrm{mod}\;2) \end{array}\right.\;\;\;\;,\;k=1,2,\ldots \end{array}$$

*Thus, S outputs a new sample of* q *iff the previous sample of* q *is matched by the previous sample mod2 of* y. *Otherwise, it lets y(k−1)mod2 pass through.*

*Consider first the following encoder–decoder pair, designed with the aim of achieving zero-error communication while maximizing $H\left(w_{1}^{n}\right)/n=I\left(w_{1}^{n};v_{1}^{n}\right)$.*

*Encoder $E_{1}$: Let the side-information sequence *r* be binary i.i.d. and independent of *q*, with Pr{r(k)=1}=β, and:*

(61) $$\begin{array}{cc} y(0) & =r(0) \end{array}$$

(62) $$\begin{array}{cc} \;y(k) & =\left\{\begin{array}{cc} r(k) & ,\;\mathrm{if}\;w(k)=(y(k-1)\;\mathrm{mod}\;2)\\ r(k)+2 & ,\;\mathrm{if}\;w(k)\neq (y(k-1)\;\mathrm{mod}\;2) \end{array}\right.\;\;\;\;,\;k=1,2,\ldots \end{array}$$

*Decoder $D_{1}$:*

(63) $$\begin{array}{cc} v(k) & =\left\{\begin{array}{cc} y(k-1)\;\mathrm{mod}\;2 & ,\;\mathrm{if}\;y(k)\leq 1\\ (y(k-1)\;\mathrm{mod}\;2)⊕1 & ,\;\mathrm{if}\;y(k)>1 \end{array}\right. \end{array}$$

*where ⊕ is the exclusive–or binary operator. With this choice, v(k)=w(k) for k=1,2,…. In addition,*

(64) $$\begin{array}{cc} w(k) & =\left\{\begin{array}{cc} q(k) & ,\;\mathrm{if}\;r(k-1)=q(k-1)\\ r(k-1) & ,\;\mathrm{if}\;r(k-1)\neq q(k-1) \end{array}\right.\;\;\;\;,\;k=1,2,\ldots \end{array}$$

*Therefore,*

(65) $$\begin{array}{c} \mathrm{Pr}\{w(1)=1\}=\alpha \beta . \end{array}$$

*and, for k≥2,*

(66) $$\begin{array}{cc} \mathrm{Pr} & \left\{w\left(k\right)=1\right\}=\left(\alpha^{2}+{\left(1-\alpha \right)}^{2}\right)\beta +\alpha \left(1-\alpha \right) \end{array}$$

*Thus, and since α=0.9, the entropy of each w(k) is maximized by β=0.5055. However, encoder $E_{1}$ makes the samples of w interdependent, so finding the value of β that maximizes $H(w_{1}^{n})/n$ (and thus $I(w_{1}^{n};v_{1}^{n})$ as well) is more involved, and that value does not need to be the same. We have found numerically that (for n=22) the maximum of $H\left(x_{1}^{n}\right)/n=I\left(w_{1}^{n};v_{1}^{n}\right)/n$ is (approximately) 0.9941 [bits/sample], attained with β=0.503, very close to the β which maximizes $H(w_{1}^{n})/n$.*

*For later comparison, we also calculate the value of $R_{\mathrm{ITL}}^{n}$ yielded by this choice of encoder:*

(67) $$\begin{array}{c} R_{\mathrm{ITL}}^{n}\overset{\left(a\right)}{=}I\left(w_{1}^{n}\to y_{1}^{n}\right)\overset{\mathrm{Thm}.\;}{=}I\left(q_{0}^{n};y_{0}^{n}\right)=\sum_{k=0}^{n}\left(H\left(q\left(k\right)|q_{0}^{k-1}\right)-H\left(q\left(k\right)|q_{0}^{k-1},y_{0}^{n}\right)\right), \end{array}$$

*where (a) holds from Theorem 7 because $H(w_{1}^{n}|y_{0}^{n})=0$. Defining the binary random variables t(k)≜1 when (y(k)mod2)=q(k) and 0 otherwise, we obtain: -4.6cm0cm*

(68) $$\begin{array}{cc} H(q\left(k\right)|q_{0}^{k-1},y_{0}^{n})\; & =H(q\left(k\right)|q_{0}^{k-1},y_{0}^{n},t\left(k-1\right))\\ & =H\left(q\left(k\right)|q_{0}^{k-1},y_{0}^{n},t\left(k-1\right)=0\right)\mathrm{Pr}\left\{t\left(k-1\right)=0\right\}+H\left(q\left(k\right)|q_{0}^{k-1},y_{0}^{n},t\left(k-1\right)=1\right)\mathrm{Pr}\left\{t\left(k-1\right)=1\right\} \end{array}$$

(69) $$\begin{array}{cc} & \;\overset{\left(64\right)}{=}H\left(q\left(k\right)\right)\mathrm{Pr}\left\{t\left(k-1\right)=0\right\}+0\cdot \mathrm{Pr}\left\{t\left(k-1\right)=1\right\} \end{array}$$

*Thus,*

(70) $$\begin{array}{cc} R_{\mathrm{ITL}}^{n} & =H(q(k))(1-\mathrm{Pr}\{t(k-1)=0\})=H(q(k))(\alpha (1-\beta )+(1-\alpha )\beta ) \end{array}$$

(71) $$\begin{array}{cc} & =0.469\times 0.4976=0.2334\;\;\;\;\left[bits/sample\right],\; \end{array}$$

*using β=0.503.*

*The second encoder/decoder pair is set to maximize $R_{\mathrm{ITL}}^{n}$, and is defined as follows:*

*Encoder $E_{2}$:*

(72) $$\begin{array}{c} y\left(k\right)=\left\{\begin{array}{cc} 1 & ,\;\mathrm{if}\;k=0\\ w(k) & ,\;\mathrm{if}\;k\geq 1 \end{array}\right. \end{array}$$

*Thus, zero-error communication is trivially attained with the simple decoding rule:*

*Decoder $D_{2}$:*

(73) $$\begin{array}{c} v(k)=y(k),\;\;\;\;k\geq 1. \end{array}$$

*In addition, encoder $E_{2}$ yields w(k)=q(k), for k≥1. Therefore,*

(74) $$\begin{array}{cc} \frac{1}{n}I\left(w_{1}^{n}\to y_{0}^{n}\right)\overset{\mathrm{Thm}.\;}{=}R_{\mathrm{ITL}}^{n} & =\frac{1}{n}H\left(q_{1}^{n}\right)=0.469\;\;\;\;\left[bits/sample\right] \end{array}$$

*As expected, encoder $E_{2}$ yields a higher $R_{\mathrm{ITL}}^{n}$ than encoder $E_{1}$. More significant is the fact that encoder/decoder pair 2 achieves the in-the-loop capacity for this channel, since:*

(75) $$\begin{array}{c} \frac{1}{n}I\left(w_{1}^{n}\to y_{0}^{n}\right)\overset{\left(a\right)}{=}I\left(q_{1}^{n};y_{0}^{n}\right)\leq H\left(q_{1}^{n}\right) \end{array}$$

The previous example illustrates an important fact that is closely related with the motivation behind the definition of $R_{\mathrm{ITL}}^{n}$: maximizing the mutual information between the messages to be transmitted and the decoded messages (a leitmotif in traditional channel coding, wherein messages are generated outside the loop) is not suitable when messages are in the loop.

Indeed, (56) provides a mathematically precise meaning to the above observation. It reveals why maximizing $I(w_{1}^{n};y_{0}^{n},p_{1}^{n})$ does not necessarily mean maximizing $I(w_{1}^{n}\to y_{0}^{n}∥p_{1}^{n})$, since the former is the sum of backward and forward information flows (represented in green and red in Figure 8, respectively).

Finally, Theorems 7 and 8 imply that in the design of any encoder for in-the-loop messages, aiming to yield the joint probability distribution of channel input and output sequences that maximizes the directed information is of practical importance: it is necessary for achieving the highest “useful” transmission rate while minimizing the probability of error.

## 7. Concluding Remarks

The widely used data processing inequality does not hold for systems with feedback. In this work, we provided a very general *directed information* data processing inequality that is applicable to feedback systems. A key insight to be gained from this new inequality is that, for nested pairs of sequences, the further apart the signals in the feedback system are from each other, the lower is the directed information between them (measuring distance from starting to finishing sequence and in the direction of cause and effect). Thus, post processing signals within a feedback loop cannot increase the information, which is similar to the open loop case. In order to obtain these results, we considered arbitrary causal systems that are interconnected in a feedback loop, with arbitrarily distributed signals. We were able to overcome the generally non-trivial dependencies between the signals in such a scenario by establishing a family of useful Markov chains that conditionally decouple the sequences in the system. These Markov chains are useful by themselves for studies involving interconnected systems. We further used the Markov chains to derive a number of fundamental information inequalities that are applicable to signals that are entirely within feedback loops or where some signals are inside and others outside the loop. With the use of these inequalities, we were able to show that the conventional notion of channel capacity is not adequate for *in-the-loop* communications. Instead, we provided the new notion of in-the-loop channel capacity, and described a special case where it was achievable. As an additional application of our results, we discussed how they allow one to generalize two known fundamental inequalities in networked control involving directed information. We are confident that our analysis provides useful insights to understand and think about information flows in single-loop feedback systems, and that our results will serve as a toolbox for research in, e.g., networked control systems or communications within a feedback loop.

There are several future research directions stemming from this work, from which we outline the following three:

(1) Establishing whether (and under which conditions, if any) in the system of Figure 1, each of the following inequalities is true or false:
  (76) $$\begin{array}{c} I\left(r^{k};e^{k}\right)\geq I\left(r^{k};x^{k}\right)\geq I\left(r^{k};y^{k}\right)\geq I\left(r^{k};u^{k}\right). \end{array}$$
(2) Extending Theorems 1 and 2 to scenarios with more than one feedback loop.
(3) Exploring if tree codes [43] can be tailored to maximize the ITL data rate instead of the conventional data rate within a feedback loop. If such adaptation is possible, it would be interesting to assess how close to the ITL channel capacity such codes can perform.

## Appendix A. Proofs

**Proof of Theorem 2.** If (q,s)⫫(r,p) and q⫫s, then (6) follows by applying Theorem 5 and then Theorem 6. If (p,s)⫫(r,q) and p⫫s, then one arrives at (6) by applying Theorem 6 followed by Theorem 5. To prove the second part, notice that:

(A1) $$\begin{array}{c} I\left(x^{k}\to y^{k}\|q^{k}\right)=I\left(x^{k}\to y^{k}|q^{k}\right) \end{array}$$

which follows since $x^{i-d_{3}\left(i\right)},y^{i}$ are deterministic functions of $\left(r^{k},p^{k},s^{i},q^{i}\right)$ and $q_{i+1}^{k}↔q^{i}↔\left(r^{k},p^{k},s^{i}\right)$, a Markov chain that results from combining $q_{i+1}^{k}↔q^{i}↔s^{i}$ with $\left(q^{k},s^{k}\right)⫫\left(r^{k},p^{k}\right)$. On the other hand, the fact that $\left(r^{k},p^{k}\right)⫫\left(q^{k},s^{k}\right)$ allows one to obtain from Theorem 1 that:

(A2) $$\begin{array}{c} I\left(x^{k}\to y^{k}|q^{k}\right)=I\left(r^{k},p^{k};y^{k}|q^{k}\right). \end{array}$$

But:

(A3) $$\begin{array}{cc} I(r^{k},p^{k};y^{k}|q^{k})\; & \overset{\left(22\right)}{=}I\left(r^{k},p^{k}\;;\;u^{k},y^{k}|q^{k}\right)-I\left(r^{k},p^{k};u^{k}|q^{k},y^{k}\right)\\ & \overset{\left(a\right)}{=}I\left(r^{k},p^{k}\;;\;u^{k},y^{k}|q^{k}\right)\\ & \overset{\left(22\right)}{=}I\left(r^{k},p^{k}\;;\;u^{k},y^{k},q^{k}\right)-I\left(r^{k},p^{k}\;;\;q^{k}\right)\\ & \overset{\left(b\right)}{=}I\left(r^{k},p^{k}\;;\;u^{k},y^{k},q^{k}\right)\\ & \overset{\left(22\right)}{=}I\left(r^{k},p^{k}\;;\;u^{k}\right)+I\left(r^{k},p^{k}\;;\;y^{k},q^{k}|u^{k}\right) \end{array}$$

where (a) is due to the fact that $u^{k}$ is a deterministic function of $q^{k},y^{k}$. Equality (b) holds if and only if (r,p)⫫q. The fact that $\left(r^{k},p^{k}\right)⫫\left(q^{k},s^{k}\right)$ allows one to obtain from Theorem 1 that $I\left(x^{k}\to u^{k}\right)=I\left(r^{k},p^{k};u^{k}\right)$. Substituting this into (A3) and then into (A2) and the latter into (A1), we obtain $I\left(x^{k}\to y^{k}\|q^{k}\right)\geq I\left(x^{k}\to u^{k}\right)$, which combined with Theorem 5 yields (7). This completes the proof. □

**Proof of Theorem 3.** Apply the chain-rule identity (22) to the *right-hand side* (RHS) of (5) to obtain:

(A4) $$\begin{array}{c} I\left(\theta^{k};y^{k}\right)=I\left(p^{k},q^{k},r^{k};y^{k}\right)=I\left(p^{k},q^{k};y^{k}|r^{k}\right)+I\left(r^{k};y^{k}\right). \end{array}$$

Now, applying (22) twice, one can express the term $I(p^{k},q^{k};y^{k}|r^{k})$ as follows:

(A5) $$\begin{array}{cc} I(p^{k},q^{k};y^{k}|r^{k})\; & =I\left(p^{k},q^{k}\;;\;y^{k},r^{k}\right)-I\left(p^{k},q^{k};r^{k}\right)=I\left(p^{k},q^{k}\;;\;y^{k},r^{k}\right)\\ & =I\left(p^{k},q^{k};y^{k}\right)+I\left(p^{k},q^{k};r^{k}|y^{k}\right), \end{array}$$

where the second equality follows since $\left(p^{k},q^{k}\right)⫫r^{k}$. The result then follows directly by combining (A5) with (A4) and (5). □

**Proof of Theorem 4.** Since q⫫(r,p,s),

(A6) $$\begin{array}{cc} I(x^{k}\to y^{k})\; & \overset{\left(a\right)}{=}I\left(x^{k}\to u^{k}\right)+I\left(q^{k};y^{k}\right)+I\left(r^{k},p^{k};y^{k}|u^{k}\right) \end{array}$$

(A7) $$\begin{array}{cc} \; & \;\overset{\left(b\right)}{=}I\left(r^{k},p^{k};u^{k}\right)+I\left(q^{k};y^{k}\right)+I\left(r^{k},p^{k};y^{k}|u^{k}\right) \end{array}$$

(A8) $$\begin{array}{cc} \; & \;\overset{\left(c\right)}{=}I\left(r^{k};u^{k}\right)+I\left(p^{k};u^{k}|r^{k}\right)+I\left(q^{k};y^{k}\right)+I\left(r^{k},p^{k};y^{k}|u^{k}\right), \end{array}$$

where (a) is due to Theorem 6, (b) follows from Theorem 1 and the fact that (s,q)⫫(r,p), and (c) follows from the chain rule of mutual information. For the second term on the RHS of the last equation, we have:

(A9) $$\begin{array}{cc} I(p^{k};u^{k}|r^{k})\; & \overset{\left(a\right)}{=}I\left(p^{k};u^{k}|r^{k}\right)+I\left(p^{k};r^{k}\right)=I\left(p^{k};r^{k},u^{k}\right) \end{array}$$

(A10) $$\begin{array}{cc} & \;\overset{\left(b\right)}{=}I\left(p^{k};r^{k},u^{k},e^{k}\right)-I\left(p^{k};e^{k}|r^{k},u^{k}\right) \end{array}$$

(A11) $$\begin{array}{cc} & \overset{\left(c\right)}{=}I(p^{k};r^{k},u^{k},e^{k})\; \end{array}$$

(A12) $$\begin{array}{cc} & \overset{\left(d\right)}{=}I\left(p^{k};e^{k}\right)+I\left(p^{k};r^{k},u^{k}|e^{k}\right) \end{array}$$

(A13) $$\begin{array}{cc} & \;\overset{\left(e\right)}{=}I\left(p^{k};e^{k}\right)+I\left(p^{k};u^{k}|e^{k}\right)+I\left(p^{k};r^{k}|u^{k},e^{k}\right) \end{array}$$

(A14) $$\begin{array}{c} \overset{\left(f\right)}{=}I\left(p^{k};e^{k}\right)+I\left(p^{k};u^{k}|e^{k}\right), \end{array}$$

where (a) holds since r⫫p, (b), (d), and (e) stem from the chain rule of mutual information (22), and (c) is a consequence of the fact that $e^{k}=S_{1}\left(u^{k-d_{1}\left(k\right)},r^{k}\right)$. Finally, (f) is due to the Markov chain $r^{k}↔\left(u^{k},e^{k}\right)↔p^{k}$, which holds because r⫫(p,s,q) as a consequence of Lemma 2 in the Appendix (see also Figure 1a). Substitution of (A14) into (A8) yields (36), thereby completing the proof. □

**Proof of Theorem 5.** Since (p,q,r)⫫s, we can apply (11) (where now (q,r) plays the role of r), and obtain

(A15) $$\begin{array}{c} I\left(x^{k}\to y^{k}\right)\geq I\left(q^{k},r^{k};y^{k}\right). \end{array}$$

Now, we apply Theorem 1, which gives

(A16) $$\begin{array}{c} I\left(q^{k},r^{k};y^{k}\right)\geq I\left(e^{k}\to y^{k}\right), \end{array}$$

completing the proof. □

**Proof of Theorem 6.** We have that:

(A17) $$\begin{array}{cc} I(x^{k}\to y^{k})\; & \overset{\left(a\right)}{=}I\left(r^{k},p^{k},q^{k}\;;\;y^{k}\right)\\ & \overset{\left(\mathrm{22}\right)}{=}I(q^{k};y^{k})+I\left(r^{k},p^{k};y^{k}\;|\;q^{k}\right)\\ & \overset{\left(A3\right)}{=}I(r^{k},p^{k};u^{k})+I\left(r^{k},p^{k};y^{k}\;,\;q^{k}\;|\;u^{k}\right) \end{array}$$

(A18) $$\begin{array}{c} \;\overset{\left(22\right)}{=}I\left(q^{k};y^{k}\right)+I\left(r^{k},p^{k};u^{k}\right)+I\left(r^{k},p^{k};y^{k}|u^{k}\right)+I\left(r^{k},p^{k};q^{k}|u^{k}|y^{k}\right) \end{array}$$

(A19) $$\begin{array}{c} \;\overset{\left(b\right)}{\geq }I\left(q^{k};y^{k}\right)+I\left(x^{k}\to u^{k}\right)+I\left(r^{k},p^{k};y^{k}|u^{k}\right)+I\left(r^{k},p^{k};q^{k}|u^{k}|y^{k}\right) \end{array}$$

(A20) $$\begin{array}{c} \;\overset{\left(b\right)}{\geq }I\left(q^{k};y^{k}\right)+I\left(x^{k}\to u^{k}\right)+I\left(r^{k},p^{k};y^{k}|u^{k}\right) \end{array}$$

where (a) follows from Theorem 1 and the assumption (r,p,q)⫫s, (b) is from Theorem 1, with equality iff (q,s)⫫(r,p), and from Lemma 2 (in the Appendix), (c) turns into equality if q⫫(r,p,s). This completes the proof. □
